# Nitrogen reduces calcium availability by promoting oxalate biosynthesis in apple leaves

**DOI:** 10.1093/hr/uhae208

**Published:** 2024-07-30

**Authors:** Yue Xing, Zi-Quan Feng, Xin Zhang, Hong-Xing Cao, Chun-Ling Liu, Han-Han Qin, Han Jiang, Zhan-Ling Zhu, Shun-Feng Ge, Yuan-Mao Jiang

**Affiliations:** College of Horticulture Science and Engineering, Shandong Agricultural University, Tai’an 271018, Shandong, China; Apple Technology Innovation Center of Shandong Province, Tai’an, 271018, Shandong, China; College of Horticulture Science and Engineering, Shandong Agricultural University, Tai’an 271018, Shandong, China; Apple Technology Innovation Center of Shandong Province, Tai’an, 271018, Shandong, China; 421 laboratory, Xinlianxin Chemical Group Co., Ltd, Henan, China; College of Horticulture Science and Engineering, Shandong Agricultural University, Tai’an 271018, Shandong, China; Apple Technology Innovation Center of Shandong Province, Tai’an, 271018, Shandong, China; College of Horticulture Science and Engineering, Shandong Agricultural University, Tai’an 271018, Shandong, China; Apple Technology Innovation Center of Shandong Province, Tai’an, 271018, Shandong, China; College of Horticulture Science and Engineering, Shandong Agricultural University, Tai’an 271018, Shandong, China; Apple Technology Innovation Center of Shandong Province, Tai’an, 271018, Shandong, China; College of Horticulture Science and Engineering, Shandong Agricultural University, Tai’an 271018, Shandong, China; Apple Technology Innovation Center of Shandong Province, Tai’an, 271018, Shandong, China; College of Horticulture Science and Engineering, Shandong Agricultural University, Tai’an 271018, Shandong, China; Apple Technology Innovation Center of Shandong Province, Tai’an, 271018, Shandong, China; College of Horticulture Science and Engineering, Shandong Agricultural University, Tai’an 271018, Shandong, China; Apple Technology Innovation Center of Shandong Province, Tai’an, 271018, Shandong, China; College of Horticulture Science and Engineering, Shandong Agricultural University, Tai’an 271018, Shandong, China; Apple Technology Innovation Center of Shandong Province, Tai’an, 271018, Shandong, China

## Abstract

N and Ca are essential nutrients for apple growth and development. Studies have found that Ca content was not low under high N conditions but was poorly available. However, the underlying physiological mechanism through which N regulates Ca availability remains unclear. In this study, apple plants were supplied with N and Ca to analyse the content, in situ distribution, and forms of Ca using noninvasive micro-test technique, electron probe microanalysis, Fourier transform infrared spectroscopy, and transcriptome analysis. A potential interaction was observed between N and Ca in apple leaves. The application of high N and Ca concentration led to a CaOx content of 12.51 g/kg, representing 93.54% of the total Ca in the apple leaves. Electron probe microanalysis revealed that Ca deposited in the phloem primarily existed as CaOx rhombus-shaped crystals. Additionally, high N positively regulated oxalate accumulation in the leaves, increasing it by 40.79 times compared with low N concentration. Specifically, N induced oxalate synthesis in apple leaves by upregulating the *MdICL*, *MdOXAC*, and *MdMDH* genes, while simultaneously inhibiting degradation through downregulation of the *MdAAE3* gene. Transcriptome and correlation analyses further confirmed oxaloacetate as the precursor for the synthesis of CaOx crystals in the apple leaves, which were produced via the ‘photosynthesis/glycolysis -oxaloacetate -oxalate -CaOx’ pathway. WGCNA identified potential regulators of the CaOx biosynthesis pathway triggered by N. Overall, the results provide insights into the regulation of Ca availability by N in apple leaves and support the development of Ca efficient cultivation technique.

## Introduction

China is the country with the largest apple production in the world, with over 30 million tons produced each year [61]. Apple cultivation plays an extremely important role in increasing the income of local farmers [1, 2]. The most significant commercial loss in fruit tree production is attributed to physiological disorders, primarily caused by inappropriate mineral nutrition [3]. However, during the actual cultivation, farmers often apply large amounts of N to increase yield and gain more benefits [4], ignoring the role of Ca, and the imbalance between the contents of N and Ca has become an important factor limiting apple quality [5]. A growing body of evidence suggests that N and Ca may act synergistically at a range of concentrations in nectarine [6], peanut [60], and populus plants [7]. Further, under high N condition, Ca content is generally high in fruit trees but is poorly available [8]. We speculate that large amounts of N disturb the balance between Ca forms within plants.

Ca is essential for plant development and maintenance, but its mobility in plants is limited, and current Ca supplementation is not always effective [9]. Studies have shown that the Ca content in apple fruit affected by bitter pit fruit is not lower than that in healthy fruit [3] and the total Ca content in plants may not be the primary factor contributing to Ca deficiency diseases, as certain forms of this nutrient may be closely associated with its availability [10, 11]. In relation to the bioavailability of Ca, trees primarily transport Ca upward in an ionic state as well as in the forms of Ca malate and Ca citrate [12]. However, Ca easily precipitates into insoluble salts and becomes fixed, which makes its transfer and utilization more difficult [13, 14]. Plants contain four main forms of Ca: (i) calcium oxalate (CaOx), which, acting as a reservoir, helps maintain an optimal ionic environment [15–17]; (ii) Ca pectin, which preserves the cell wall structure; (iii) water-soluble Ca, which functions as a secondary messenger signal; and (iv) Ca phosphate, which plays a role in energy metabolism [12, 18]. More than 90% of the Ca in plants is present in the form of CaOx crystals [17, 19]. Plants with a high CaOx content, such as spinach, have poor Ca bioavailability compared to plants with a low CaOx content, such as kale [20, 21]. The formation of insoluble CaOx crystals is associated with high-capacity Ca regulation or sequestration, ion balance, detoxification from oxalate, photosynthesis, and plant protection [17, 22]. Therefore, precise control of the Ca concentration in plants is essential by regulating CaOx accumulation in plants.

Oxalate is an end product of the carbon metabolism connected to photosynthesis in plants [23–25]. It has been reported that CaOx crystals were produced in photosynthetic palisade cells. The biosynthesis of oxalate in plants includes three documented pathways: the glyoxylate pathway, the ascorbate pathway, and the oxaloacetate pathway [26, 27, 28]. To date, studies have shown that oxalate accounts for approximately 5% of the total organic acid in apple fruit [29]. It is usually possible to manipulate oxalate metabolism by using agricultural practices or plant biotechnology. Several studies have investigated the significant accumulation of oxalate under exposure to exogenous nitrate conditions in rice [30, 31], spinach [20], maize [32], and rhubarb [27]. Additionally, variations in oxalate concentrations would inevitably affect the Ca content by the form of CaOx in plants [22, 24]. However, the mechanism for how N and Ca regulate oxalate content and affect Ca availability in apple plants has rarely been investigated.

Here, apple leaves were characterized using non-invasive micro-test (NMT), scanning electron microscopy X-ray microanalysis, Fourier transform infrared spectroscopy (FTIR), and RNA-Seq to investigate the accumulation, *in vivo* forms, and the spatial localization of Ca under high N condition. The objectives of this study were (i) to identify the phenotypic changes in apple leaves under the synergistic supply of N and Ca; (ii) to analyse Ca content, forms, and in situ localization under N supply; (iii) to detect the main form of CaOx crystals and determine the oxalate content in apple leaves under N supply; (iv) to reveal the regulatory pathways underlying the CaOx biosynthesis in apple leaves under N supply; and (v) to identify potential regulators of the CaOx biosynthesis pathway triggered by N. This information will assist in improving Ca availability and in the breeding of highly Ca-efficient apple rootstocks.

## Results

### Phenotype and growth in leaves

**Figure 1:**
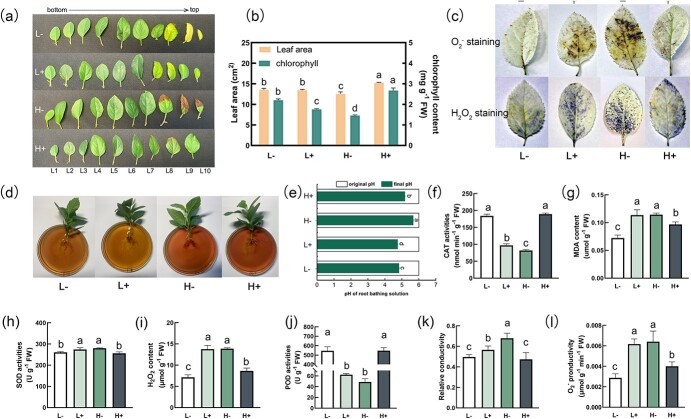
Effects of N and Ca supply on phenotype and antioxidant metabolism in apple leaves. L−: low N without Ca (0.5 mM NO_3_^−^ + 0 mM Ca), L+: low N with Ca (0.5 mM NO_3_^−^ + 5 mM Ca), H−: high N without Ca (10 mM NO_3_^−^ + 0 mM Ca), and H+: high N with Ca (10 mM NO_3_^−^ + 5 mM Ca). **(a)** Leaf phenotype; **(b)** leaf area and chlorophyll content; **(c)** leaf staining with Nitro-blue tetrazolium (NBT) and 3, 3′-Diaminobenzidine (DAB); **(d)** hydrogen ion secretion in root; **(e)** rhizosphere pH value; **(f)** catalase (CAT) activity; **(g)** maleic dialdehyde (MDA) activity; **(h)** superoxide dismutase (SOD) activity; **(i)** H_2_O_2_ activity; **(j)** peroxidase (POD) activity; **(k)** relative conductivity; **(l)** O_2_^−^ productivity.

Under low N conditions, low Ca caused new leaf yellowing compared with sufficient Ca. However, under high N conditions, leaf burning was observed for new leaves, suggesting that high N aggravated Ca deficiency. This was supported by the fact that increasing Ca application alleviated high N-induced Ca deficiency (Fig. 1a). Under high N conditions, sufficient Ca resulted in higher chlorophyll content and larger leaf area than low Ca conditions (Fig. 1b). Furthermore, high N resulted in a significant increase in the rhizosphere pH staining, particularly when Ca was deficient (Fig. 1d; Fig. S7, see online supplementary material).

NBT and DAB staining were used to determine whether reactive oxygen species (ROS) were generated in the leaves (Fig. 1c). Apple leaves exhibited a considerably stronger color intensity under L+ and H− treatments. Under low N conditions, sufficient Ca caused an increased accumulation of ROS, but this process was inhibited under high N conditions. Consistent with the antioxidant phenotype, the contents of O_2_^−^, H_2_O_2_, and MDA, as well as the relative conductivity were significantly increased under L+ and H− treatments. Simultaneously, the activities of CAT and POD were significantly decreased. This was supported by the fact that there was a correlation between low levels of N and Ca, as well as high levels of N and Ca, indicating that the balance between N and Ca levels ensures optimal plant growth.

### N and Ca contents and in situ distribution

**Figure 2:**
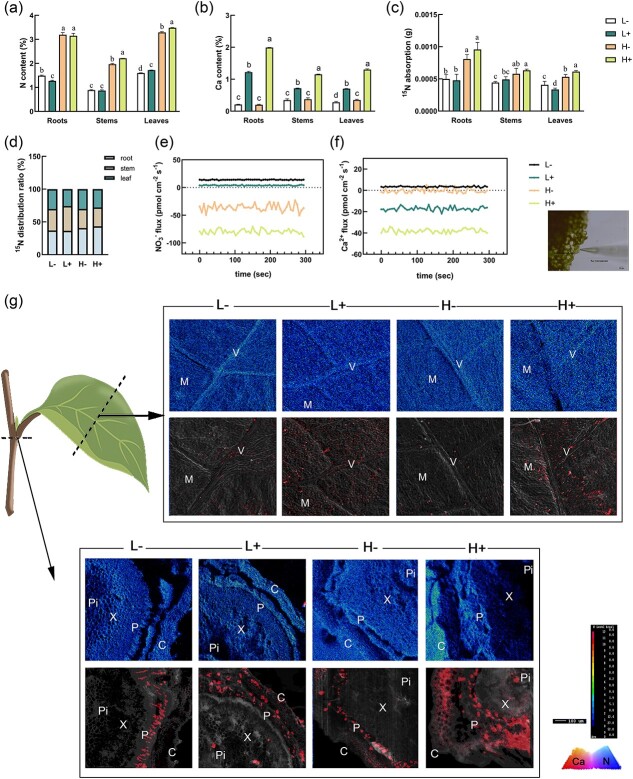
Contents and localization of N and Ca. **(a)** N content in plants; **(b)** Ca content in plants; **(c)** 15 N uptake; **(d)**  ^15^N allocation rate; **(e)** NO_3_^−^ flow rate in leaf mesophyll cells; **(f)** Ca^2+^ flow rate and real-time electrode images of leaf mesophyll cells; **(g)** schematic showing sampling sites. *In situ* localization of N and Ca in leaves and cross section of the stem node. The secondary electron image is combined with the image of the localization of the X-ray Ca spectrum signal. The densities of N and Ca are represented by the color card and red dots, respectively. C, cortex; M, mesophyll cell; P, phloem; Pi, pith; V, vascular bundle; X, xylem. Scale =100 μm.

The N content in the leaves was highest and lowest under H+ and L− treatments, respectively (Fig. 2a). Under sufficient Ca conditions, low N caused a lower Ca accumulation in all of the organs, and high N caused significantly higher Ca (Fig. 2b). It was shown that N acts synergistically with Ca in this concentrations range. Analysis using a ^15^N isotope tracer further supported these findings, showing an increased ^15^N distribution in leaves under high N conditions (Fig. 2c and d). The ion flux generally determines the N and Ca content in plants. To corroborate these results, NMT was used to measure the net flow rates of NO_3_^−^ and Ca^2+^ in mesophyll cells, high N caused net influx rate of Ca^2+^ higher in mesophyll cells compared with low N, while Ca^2+^ was efflux under low N conditions (Fig. 2f).

The N and Ca distribution in apple leaves (Fig. 2g) and stem cross-section tissues (Fig. 2h) was analysed using scanning electron microscopy combined with a Ca electron probe. N was uniformly distributed in the leaves and stem cross-sections, with stronger signals detected under high N conditions. However, Ca was unevenly distributed within the leaves, primarily accumulating along the leaf veins as these are Ca-rich areas. In the stem cross-sections, Ca primarily accumulated in the phloem and cortex regions; however, Ca was less present within the xylem. For their distribution in the phloem, Ca was sporadically detected under low N condition in the leaves and stem cross-sections. Under high N conditions, the presence of Ca-rich areas was more pronounced, particularly within the pith and cortex regions. In summary, N and Ca appeared to exert a synergistic effect within a specific range of concentrations, with Ca exhibiting a larger distribution in the phloem under high N conditions.

### Ca forms and micro-morphology of CaOx crystals in apple leaves

**Figure 3:**
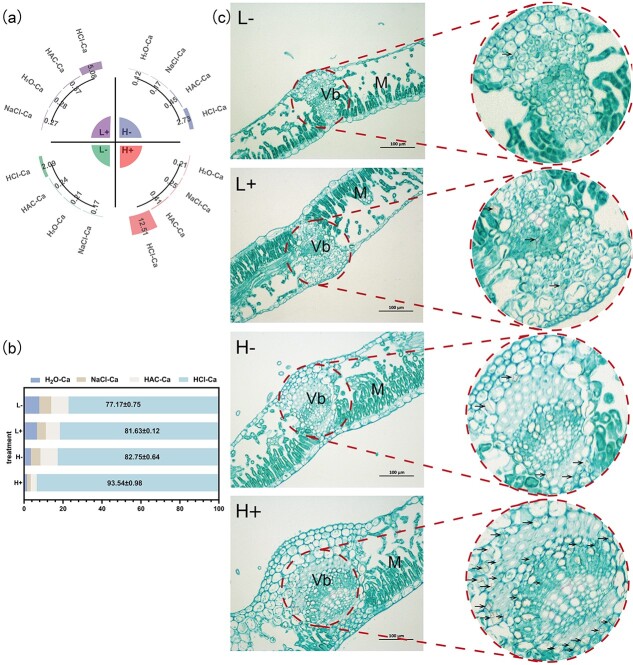
Qualitative and quantitative data related to Ca forms and the morphology and distribution of CaOx crystals in the apple leaves. **(a)** The content of Ca forms in apple leaves; **(b)** proportions of Ca forms present in apple leaves; **(c)** microscopy images of a leaf tissue section. Low magnification is shown on the left (scale = 100 μm), double magnification is shown on the right, and black arrow indicates CaOx crystals. M, leaf mesophyll; V, vascular bundle.

The quantitative analysis of the Ca forms in the leaves revealed that CaOx was the most abundant form present under all four treatments, NaCl-Ca was the least abundant under low N conditions, and H_2_O-Ca exhibited the lowest concentrations under high N conditions. Additionally, under high N conditions, sufficient Ca caused the CaOx content to reach 12.51 g/kg, which was 2.46 times and 4.58 times higher than those observed under the L+ and H− treatments, respectively (Fig. 3a). The analysis of the Ca form ratios indicated that approximately 70%–90% of Ca in apple leaves was stored as CaOx, with a notable proportion of 93.54% being observed under the H+ treatment (Fig. 3b). Furthermore, optical microscopy observations revealed that crystals were primarily enriched within the vascular bundles of the phloem but were not observed in the xylem (Fig. 3c). Under high N conditions, sufficient Ca caused the density of crystals to increase, with most crystals exhibiting a rhombus-like shape, which were present in the phloem within the vascular bundle of apple leaves (black arrow).

### Oxalate content in leaves

**Figure 4:**
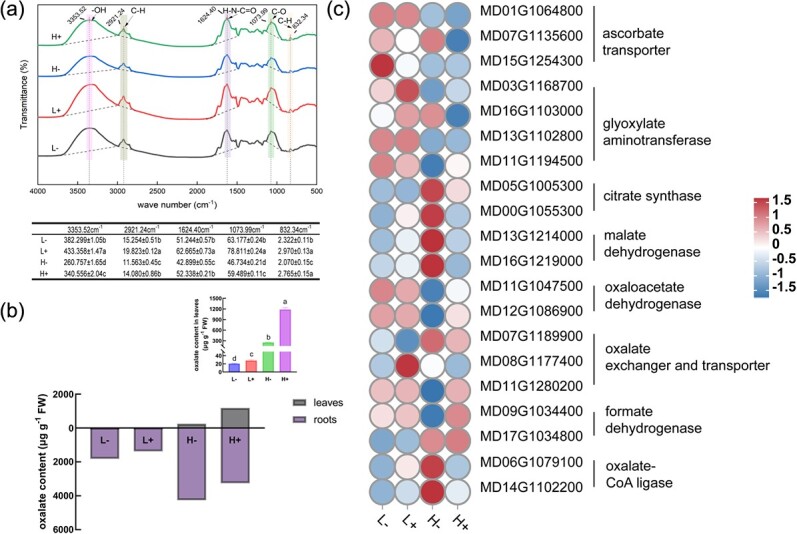
**(a)** FTIR spectroscopy of the apple leaves under the (i) L−, (ii) L+, (iii) H−, (iv) H+ treatments; **(b)** HPLC chromatograms and absorption peak areas; **(c)** plant oxalate content; **(d)** the curves shown in the plot represent power functions of N and oxalate contents.

FTIR spectroscopy was used to discriminate and identify the biomolecules present in apple leaves supplied with N and Ca (Fig. 4a). Figure 4a shows that the waveforms, peak numbers, and peak shapes of the infrared spectra were similar at various wavelengths, and differences were detected in the peak intensity. Specifically, at 3353.52 cm^−1^, the peak value decreased under high N conditions but increased under sufficient Ca conditions. This suggested that under low N conditions, sufficient Ca caused that the contents of organic substances, such as sugars and amino acids, which function as osmotic regulators, increased. The absorption peak at 2921.24 cm^−1^ was associated with the stretching vibration of the carboxylic acid O-H and methyl C-H bonds, which were primarily formed in vitamins, various membrane, and cell wall tissue components and were related to transport. The peak value of Ca^2+^ decreased under high N conditions, indicating that the organic acids secreted by leaves chelate Ca^2+^, which results in a reduction in the carboxylic acid O-H bonds. Under low N conditions, there was a lack of chelation by the organic acid secreted by the leaves and a weakening of its chelating force. The increase of the Ca^2+^ peak at 1624.40 cm^−1^ may be related to an increase in amino acids, peptides, and proteins in the leaves, while its decrease under high N conditions may be linked to an excess of carboxylate production. The variation of the peak value at 1060 cm^−1^ was mainly because of membrane lipid peroxidation, which decreased under high N conditions.

**Figure 5:**
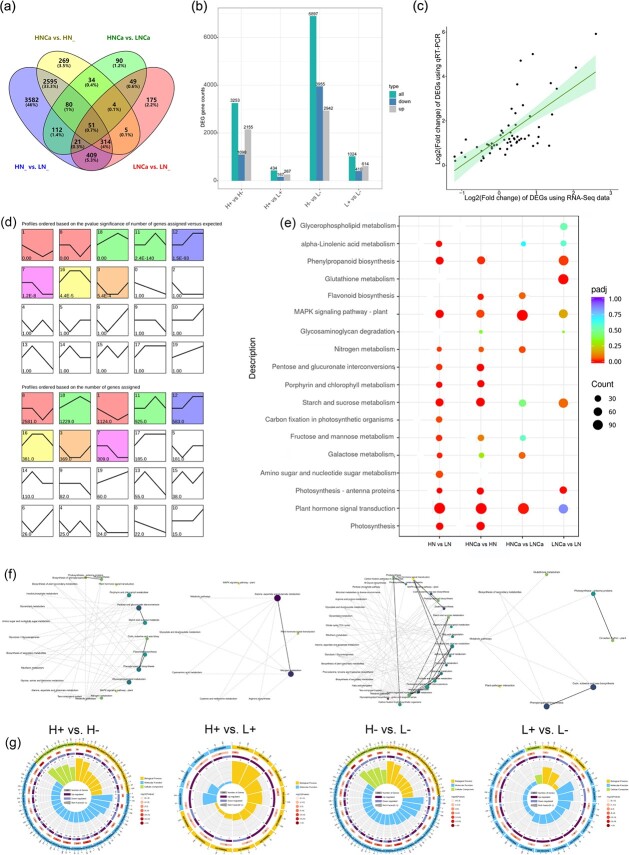
RNA-Seq identification and functional enrichment analysis of the Gene Ontology (GO) and Kyoto Encyclopedia of Genes and Genomes (KEGG) of DEGs in apple leaves under N and Ca conditions. **(a)** Venn diagram of DEGs; **(b)** number of DEGs between different comparison groups; **(c)** the RNA-Seq and qRT-PCR results were subjected to correlation analysis based on 10 selected genes; **(d)** cluster analysis of 11 608 DEGs based on the K-means method; **(e)** KEGG analysis of DEGs in leaves under N and Ca conditions; **(f)** KEGG pathway (solid line) and associated pathway (dashed line); **(g)** GO enrichment analysis. The enrichment circle is divided into four circles from the outside to the inside. The first circle indicates the classification of enrichment, and the outside circle represents the coordinate scale of gene number. The color code in the second circle defines the *P*-value size, the number is the classification in the background gene; the third circle is a bar chart of the proportions of upregulated and downregulated genes; the fourth circle indicates the enrichment factor values.

Oxalate is the simplest of the dicarboxylic acids and in this study, its content in apple leaves was further determined using HPLC chromatography (Fig. 4b). The results showed that the oxalate content significantly increased under high N conditions, reaching its highest level under H+ treatment, which resulted in contents that were 57.42, 41.79, and 4.68 times higher than those obtained under the L−, L+, and H− treatments, respectively (Fig. 4c). A strong positive correlation was detected between the oxalate concentration and N content (r^2^ = 0.86).

### Identification and functional enrichment of DEGs

DEGs were determined based on the criteria of FC ≥ 2 |log2FoldChange| > 1 and padj <0.05, with an FDR <0.01 multiple change standard. In the comparisons between treatments, i.e., H+ vs. H−, H+ vs. L+, H− vs. L−, and L+ vs. L−, a total of 3253 DEGs (2155 upregulated and 1098 downregulated), 434 DEGs (267 upregulated and 167 downregulated), 6897 DEGs (2942 upregulated and 3955 downregulated), and 1024 DEGs (614 upregulated and 410 downregulated) were identified, respectively. Venn diagrams revealed common sets of 51 DEGs shared among all four groups, which were involved in various biological processes, including the response to acid chemical (GO:0001101), organic acid biosynthetic process (GO:0016053), and dicarboxylic acid metabolic process (GO:0043648). To validate the reliability of the RNA-Seq data obtained in this study, 10 representative DEGs were selected for qRT-PCR (Table S1, see online supplementary material). The results showed a linear Pearson’s (r = 0.7) regression between RNA-Seq and qRT-PCR data (Fig. 5c), further supporting the accuracy of our RNA-Seq analysis.

A total of 11 608 DEGs were identified and subsequently classified into six groups based on their size and quantity using K-means clustering (Fig. 5d). The red cluster, which comprised 3705 DEGs, exhibited a significant decrease in expression under the H− treatment, while the green cluster, which included 2154 DEGs, exhibited the highest level of expression under the H− treatment.

GO enrichment analysis was used to further annotate the DEGs in terms of molecular functions, cellular components, and biological processes (Fig. 5g). The enriched GO terms were identified using a *P*-value threshold < 0.05. The DEGs in the molecular function category were compared among the four treatment groups. The results showed that in the H+ vs. H− comparison, most DEGs were enriched in hydrolase activity, acting on glycosyl bonds (GO:0016798), xyloglucan: xyloglucosyl transferase activity (GO:0016762), glucosyltransferase activity (GO:0046527), fructose 1,6-bisphosphate 1-phosphatase activity (GO:0042132), carbohydrate phosphatase activity (GO:0019203), and oxidoreductase activity (GO:0016620). In H+ vs. L+, the DEGs were mainly enriched in carbohydrate binding (GO:0030246), fructose 1,6-bisphosphate 1-phosphatase activity (GO:0042132), calcium ion binding (GO:0005509), and antioxidant activity (GO:0016209). In H− vs. L−, they were enriched in hydrolase activity, acting on glycosyl bonds (GO:0016798), xyloglucan: xyloglucosyl transferase activity (GO:0016762), carbohydrate binding (GO:0030246), fructose 1,6-bisphosphate 1-phosphatase activity (GO:0042132), and calcium ion binding (GO:0005509). Finally, in L+ vs. L−, the enriched pathways were carbohydrate binding (GO:0030246), oxidoreductase activity (GO:0016682), hydrolase activity, and acting on glycosyl bonds (GO:0016798).

As for the KEGG enrichment analysis, in the H+ vs. L+, H+ vs. H−, H− vs. L−, and L+ vs. L− comparisons, a total of 26, 17, 9, and 6 KEGG pathways, respectively, were found to be significantly enriched. These pathways included ‘photosynthesis (mdm00195)’, ‘plant hormone signal transduction (mdm04075)’, ‘starch and sucrose metabolism (mdm00500)’, ‘fructose and mannose metabolism (mdm00051)’, ‘carbon metabolism (mdm01200)’, ‘citrate cycle (mdm00020)’, and ‘phenylalanine metabolism (mdm00360)’ (Fig. 5e). It is worth noting that, in H+ vs. H−, ‘photosynthesis’, ‘plant hormone signal transduction’, ‘2-oxocarboxylic acid metabolism’, and ‘starch and sucrose metabolism’ were significantly enriched. Overall, the results showed that ‘photosynthesis’, ‘TCA cycle’, ‘glucose metabolism’, and ‘glyoxylate and dicarboxylate metabolism’ were regulated by the supply of N and Ca.

### Determination of the pathways of oxalate synthesis under N stress in apple leaves

CaOx crystals are considered as an internal CO_2_ source in plants and are referred to as ‘alarm photosynthesis’ [25]. The accumulation and regulation of oxalate content indirectly rely on photosynthesis, and it has been reported that this compound can also be directly produced from glycolysis and the TCA cycle to compensate for oxalate. Current studies have identified three pathways for the biosynthesis of oxalate in plants: the glyoxylate pathway, ascorbate pathway, and oxaloacetate pathway. Therefore, based on previous research, a network of metabolism pathways for the biosynthesis of CaOx and oxalate was here constructed.

**Figure 6:**
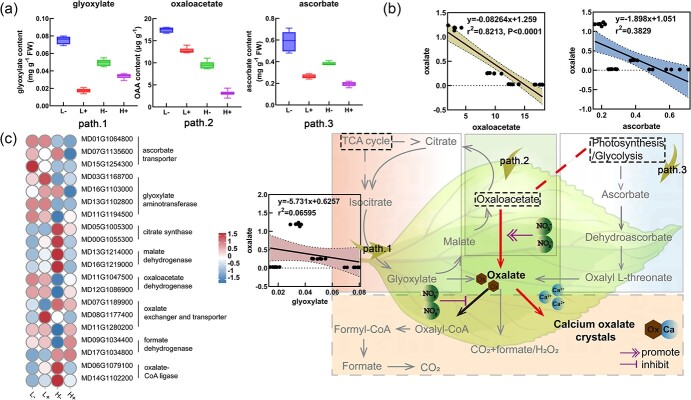
**(a)** Contents of glyoxylate, oxaloacetate, and ascorbate as biosynthetic precursors of oxalate. **(b)** Pearson correlation between oxalate and its biosynthetic precursors, and schematic model of CaOx biosynthesis and degradation in plants (the pathway of CaOx biosynthesis under N stress is outlined in red, while the black line in bold denotes the degradation pathway). **(c)** Gene expression in the transcriptome during oxalate synthesis, degradation, and transport. The red and blue colors in the heat map indicate upregulation and downregulation, respectively.

The content of ascorbate and glyoxylate—precursors of oxalate synthesis in apple leaves—was determined using liquid chromatography (Fig. 6a). The contents of ascorbate and glyoxylate were highest under L− treatment, which were 1.64 and 1.66 times greater than those of H− treatment, respectively. The oxaloacetate content was highest in the L− treatment and lowest in the H+ treatment, which is contrary to the observed oxalate content trend. Pearson correlation analysis of oxalate and ascorbate contents in apple leaves showed no correlation (y = −1.898x + 1.051, r^2^ = 0.3829), nor between the oxalate and glyoxylate contents (y = −0.4602x + 0.06449, r^2^ = 0.2118). In contrast, a very significant positive correlation was observed between oxalate and oxalacetate contents (y = −0.08264x + 1.259, r^2^ = 0.8213), indicating that oxalacetate is an effective precursor of oxalate synthesis in apple leaves under high N conditions (Fig. 6b).

The oxalate content in the leaves was shown to be influenced by biosynthesis and degradation. To further investigate this, 20 genes involved in oxalate synthesis, degradation, and transport were analysed (Fig. 6c). After treatment with N and Ca, there were no significant differences observed in the expression levels of the ascorbate transporter and glyoxylate aminotransferase. Under high N conditions, low Ca caused upregulation of citrate synthetase and malate dehydrogenase (*MDH*), but sufficient Ca downregulated these. However, under high N conditions, low Ca significantly downregulated oxaloacetate hydrolase (*OXAC*) gene. Additionally, under low Ca conditions, high N led to a significant upregulation of oxalyl-CoA synthetase (*AAE3*), with a 4.19-fold increase in expression compared to the level observed under low N conditions, which corresponded to the observed variation in oxalate content. The change in formate dehydrogenase (*FDH*) was not significant in all four treatments. Consequently, it was concluded that the oxaloacetate pathway is responsible for the oxalate biosynthesis under high N conditions. Specifically, N was shown to induce oxalate production by upregulating isocitrate lyase (*ICL*), *OXAC*, and *MDH* inhibiting degradation by downregulating *AAE3*.

### Enrichment pathways related to oxalate and CaOx crystals

**Figure 7:**
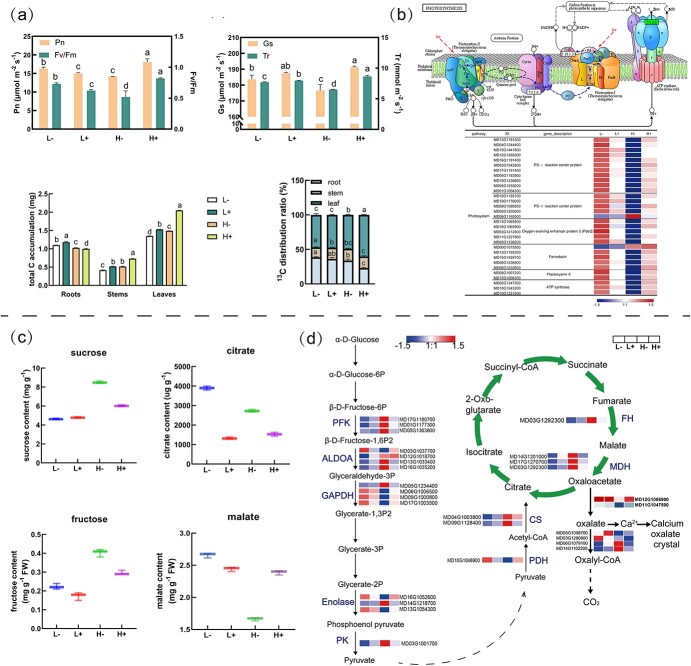
**(a)** Photosynthetic indices (*Pn*, *Tr*, and *Gs*) and *Fv/Fm* parameters in apple leaves; C accumulation and 13C allocation in plants. **(b)** Expression of DEGs involved in photosynthesis. **(c)** Sucrose, fructose, citrate, and malate contents in leaves. **(d)** Expression patterns of DEGs involved in glycolysis and the TCA cycle. ALDOA, fructose-diphosphate aldolase; FH, fumarate hydrogenase; GAPDH, glyceraldehyde-3-phosphate dehydrogenase; MDH, malate dehydrogenase; PDH, pyruvate dehydrogenase; PFK, 6-phosphofructokinase; PK, pyruvate kinase; SCS, succinyl-CoA synthetase.

The GO results showed that most photosynthesis-related terms, including ‘photosynthesis’, ‘photosystem II’, ‘photosystem I’, ‘cellular carbohydrate metabolic process photosystem’, ‘photosynthetic membrane’, and ‘thylakoid’, were significantly enriched. Under high N conditions, sufficient Ca resulted in the highest net photosynthetic rate, stomatal conductance, transpiration rate, *Fv/Fm*, and C content, whereas these parameters were the lowest under low Ca conditions (Fig. 7a). Furthermore, ^13^C isotopic tracer analysis showed that high N led to increased carbohydrate accumulation in the leaves. Interestingly, under low N conditions, low Ca resulted in the upregulation of proteins associated with photosynthetic reaction centers (*Psb* and *Psa*), plastocyanin on the thylakoid membrane, *PsbQ* enzyme, and ATP synthase genes on photosystem II. Conversely, under high N conditions, downregulation was observed under low Ca conditions, possibly indicating a plant stress response when there was an imbalance between N and Ca supply (Fig. 7b).

**Figure 8:**
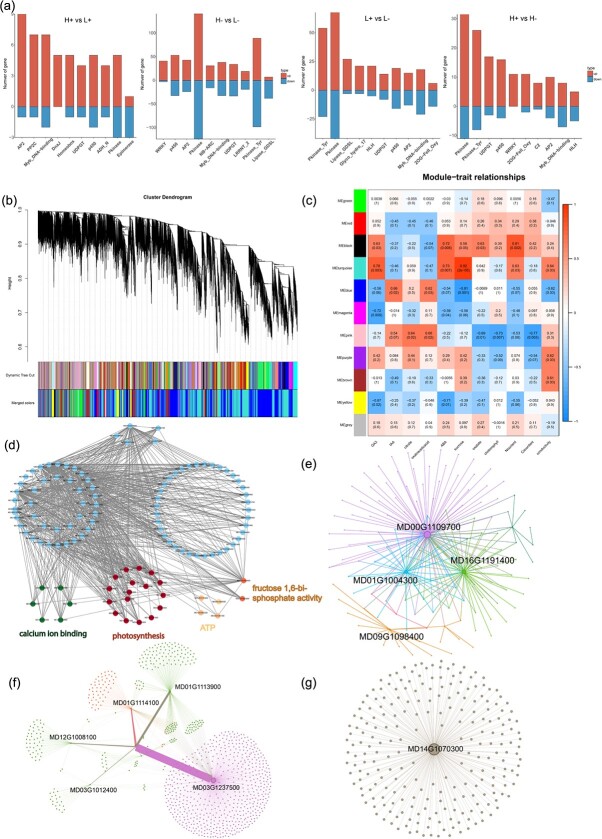
Differential expression of transcription factors and identification of oxalate-related gene co-expression modules based on WGCNA. **(a)** Number of DEGs associated with transcription factors in the comparison of the following treatments: H+ vs. L+, H− vs. L−, L+ vs. L−, and H+ vs. H−. The positive and negative values on the Y-axis indicate the number of upregulated and downregulated transcription factors, respectively. **(b)** hierarchical clustering tree showing the co-expression modules identified via WGCNA. Each leaf on the tree represents a gene, and 11 main modules labeled with different colors are visible. **(c)** Character-module association. Each row corresponds to a module, which is marked with a color. **(d)** Regulatory network of genes related to carboxylic acid metabolism in the blue module. The text represents the GO terms involved in the genes. **(e)** The top 10 most connected central genes in the blue module. **(f)** Transcriptional regulatory network of genes related to carboxylic acid metabolism in the black module. **(g)** Regulatory networks of the 10 most connected central genes in the turquoise module. Each circle represents a gene, the circle size indicates the level of the connectivity, and the core genes are labeled with text.

Carbohydrate metabolism plays a vital role in energy utilization in plants. An in-depth analysis of gene function revealed the involvement of various pathways related to photosynthesis and glucose metabolism in leaves exposed to different N and Ca conditions. After 20 days of treatment, the levels of intermediate metabolites of the TCA cycle and glycolysis were measured in the apple leaves. High N upregulated the sucrose and fructose contents compared with low N levels, especially under low Ca conditions (Fig. 7c). Low Ca exhibited significantly higher citric acid content than that of sufficient Ca. Under low N conditions, the citric acid content observed under low Ca conditions was the highest, at 2.93 times that under sufficient Ca conditions. Under low Ca conditions, however, high N caused a decrease in malate content. This decrease may be attributed to the significant upregulation of the *MDH* gene. In contrast, no significant variation in malate content was observed under sufficient Ca conditions.

The expression patterns of DEGs encoding crucial enzymes during glycolysis and the TCA cycle were examined (Fig. 7d). Under high N conditions, low Ca caused a consistent upregulation of the *PFK* gene, a key regulatory enzyme in the glycolytic pathway, compared with sufficient Ca. Similarly, genes encoding PK and citrate synthetase (CS), both critical regulatory enzymes of the TCA cycle, exhibited an analogous upregulation pattern in the leaves. Under high N conditions, low Ca caused a significant upregulation of *FH* and *MDH* compared to sufficient Ca. Under low N conditions, low Ca caused the downregulated of these genes. However, under high N conditions, low Ca upregulated oxalo-co synthetase (*OCS*) and downregulated them under sufficient Ca conditions. Overall, DEGs that were more involved in the pathways related to photosynthesis, the TCA cycle, and the glycolysis pathways exhibited an early upregulation in response to the interaction between N and Ca.

### Identification of genes related to oxalate synthesis via weighted correlation network analysis (WGCNA)

Transcription factors and protein kinases are essential for regulating gene expression and signal transduction. In our study, 759 differentially expressed transcription factors were identified, accounting for 40.49% of all DEGs (Fig. 8a). These transcription factors belonged to families such as MYB, WRKY, P450, AP2, HLH, and PP2C. These genes showed predominantly upregulated expression patterns in the +Ca vs. −Ca and HN vs. LN comparison groups.

WGCNA is a systematic biological approach for describing patterns of gene association with symptom phenotypes and searching significantly correlated genes in tissues. Here, this analysis involved genes with fragments per kilobase per million (FPKM) <1 and resulted in a total of 21 982 genes. The analysis was specifically conducted in relation to physiological characteristics (Fig. 8b). In WGCNA, modules are defined as clusters of highly correlated genes, and genes within the same module exhibit strong correlations. A total of 11 distinct modules were identified in this study (Fig. S2, see online supplementary material). Figure 8c illustrates the coefficients of correlation between characteristic genes from the different modules and various physiological parameters as well as gene abundances. Notably, the blue, black, and turquoise modules showed significant correlations with oxalate and oxaloacetate contents (Fig. S3, see online supplementary material). Subsequently, these modules were subjected to GO and KEGG enrichment analyses to visualize the regulatory relationships among genes.

The blue module, which consisted of 8179 genes, showed downregulation in the H− treatment. This module exhibited an expression pattern that was opposite to that of most other modules (Fig. 8d). A regulatory network was constructed for genes associated with the dicarboxylic acid metabolism and synthesis within this module (Fig. 9e). According to GO enrichment analysis, within this regulatory network, genes associated with the carboxylic acid metabolism genes participate in photosynthesis, Ca^2+^ binding, ATPase activity, and fructose 1,6-diphosphatase activity. KEGG analysis revealed enrichment in metabolic pathways (Fig. S4, see online supplementary material). Furthermore, the regulatory networks of the 10 most connected genes in the module were visualized, revealing potential candidate genes, such as MD00G1109700, MD16G1191400, and MD01G1004300, which encode subunits of the photosystem I reaction center, and MD09G1098400, a cell cycle regulator, all of which play a crucial role in oxalate synthesis.

The black module, which consisted of 509 genes, showed higher expression levels under the H+ and H− treatments than under the L− and L+ treatments (Fig. 8f). Therefore, it was hypothesized that the genes in this module may be associated with high N concentrations. Based on GO enrichment analysis, these genes were shown to be involved in protein dephosphorylation, catalytic activity, and catabolism (Fig. S5, see online supplementary material).

The turquoise module comprises 8,833 genes. These genes exhibited increased expression levels under the H− treatment and an expression pattern that was completely opposite to that of genes in the blue module. GO and KEGG enrichment analyses revealed that this module was involved in glycolysis, carbohydrate biosynthesis, protein transport, and other biological processes (Fig. S6, see online supplementary material). Moreover, MD14G1070300, which encodes galactose-sucrose galactosyltransferase and is primarily involved in sucrose synthesis, exhibited the highest connectivity and served as the core gene within the network (Fig. 8g). Indeed, potential regulators of the CaOx biosynthesis pathway triggered by N were identified through WGCNA.

## Discussion

N is a key nutrient that affects Ca availability. In this study, high N concentrations aggravated the imbalance between N and Ca, further exacerbating Ca deficiency symptoms under Ca-limited conditions [33]. However, Ca deficiency symptoms may be visible in the field because of the poor redistribution of Ca within plants. A growing body of evidence suggests that Ca forms are essential for plant growth and development [17, 34]. Most Ca accumulates as CaOx crystals, which account for over 90% of this nutrient in plants [16, 35]. In this study, high N was shown to positively regulated oxalate, causing it to bind with Ca to form CaOx. Under the H+ treatment, CaOx accounted for up to 93.54% of the total Ca content in apple leaves, in line with previous research [21]. The CaOx crystals can be found in all photosynthetic organisms, such as algae, lower vascular plants, gymnosperms, and angiosperms, in the forms of crystal sand, raphide, druse, styloid, and prismatic components [22]. Electron microscopy further revealed that the CaOx crystals present in the phloem of the apple leaves were crystal sand, rhomboidal, and primarily located in the phloem of vascular bundles, as similarly observed in litchi [36]. In relation to the Ca distribution, preliminary studies have determined that Ca ions in the leaves are partly dependent on imports from the xylem via the transpiration stream; however, Ca was not mobile in phloem and could not be easily retranslocated [8, 37]. However, the presence of crystals in the phloem indicated that a considerable amount of Ca was sequestered as CaOx, enabling continuous Ca accumulation in the tissue. Therefore, the formation of CaOx may be a balancing mechanism that maximizes Ca transport while preventing Ca signaling [12, 51]. These findings suggested that, along with the xylem, the phloem may also be a major pathway for Ca transport [17].

N could effectively prompt oxalate accumulation and had been reported to be correlated with organic acids; for example, high N was known to lower malate concentrations [38]. Studies have also shown that N could significantly alter the TCA cycle activity and glycolytic flux, which are closely related to organic acids [39]. In this study, citrate content decreased under high N conditions. Also, high N significantly increased the oxalate content by 40.79 times than low N under sufficient Ca conditions. The external application of N has been shown to result in a substantial buildup of oxalate in spinach and rice [18, 40]. This could be caused by the fact that N application increases oxalate biosynthesis to maintain intracellular pH homeostasis. High N resulted in a significant release of OH^−^; in order to maintain cytoplasmic pH homeostasis, cells may enhance glycolysis and the TCA cycle, thereby facilitating the synthesis of organic acids [41]. Another reason could be that N inhibited oxalate degradation and promotes oxalate synthesis, ultimately leading to oxalate accumulation. In addition, oxalate accumulation is able to trigger PCD and induce ROS production, resulting in wilted tissue and stomatal opening [28]. In this study, Ca deficiency symptoms became more severe under high N than under low N conditions, leading to a significant increase in ROS production (Fig. 1a and c), which was associated with enhanced oxalate content under the H− treatment.

The process of CaOx crystal formation consists of two steps: the *in vivo* synthesis of oxalate and the *in vitro* transport of Ca ions [42, 43]. Three pathways for the synthesis of oxalate exist in plants: the glyoxylate pathway, the ascorbate pathway, and the oxaloacetate synthesis pathway [44, 45]. Oxalate can be degraded through oxidation, decarboxylation, and acetylation processes, ultimately producing CO_2_ [15, 46]. These biosynthetic processes vary depending on the external environment and species [47, 48]. Yu *et al.* [31] found that glyoxylate is an efficient precursor for oxalate biosynthesis in rice. Moreover, in the apple leaves exposed to cold stress, the glyoxylate pathway was shown to be responsible for synthesizing oxalate [49]. In this study, oxaloacetate was identified as the precursor of oxalate synthesis in apple leaves under high N supply. This finding was corroborated by the following evidence: (i) first, oxalate accumulation increased with increasing NO_3_^−^ levels, while the content of oxaloacetate showed the opposite trend, a phenomenon that may be related to anaplerotic reactions in the TCA cycle [50]; (ii) second, Pearson correlation analysis confirmed the correlation between oxalate and each of the three precursor compounds, such as ascorbate, glyoxylate, and oxaloacetate, we found oxaloacetate showed a significant correlation with oxalate; and (iii) finally, transcriptome analysis revealed differential gene enrichment associated with photosynthesis, glycolysis, and the TCA cycle. Specifically, Ca upregulated photosynthesis, carbohydrate binding, and oxidoreductase enzyme activities by targeting multiple Ca-binding sites in plant photosystem II. High N enhanced the response to acidic chemical pathways. At the same time, genes involved in oxalate synthesis, such as *MdICL* and *MdMDH*, as well as those responsible for oxalate degradation, such as *MdAAE3*, were upregulated under the H− treatment, and the expression of oxalate hydrolase was downregulated. Therefore, this evidence supports the hypothesis that the oxaloacetate pathway plays a crucial role in oxalate biosynthesis and that acetylation is the primary mechanism for oxalate degradation. Thus, it can be concluded that the ‘photosynthesis/glycolysis – oxaloacetate – oxalate – CaOx’ pathway is activated under high N conditions. Consistent with our results, transcriptome studies have revealed that elevated oxalate levels in spinach are associated with specific gene subsets involved in pathways related to oxaloacetate degradation, including *OXAC*, malate synthetase (*MLS*), and *MDH* [51, 52]. However, it remains to be explored whether other stressors, in addition to N stress, affect different CaOx biosynthesis pathways in apple plants.

WGCNA is an effective method for identifying the clustering of genes with similar expression patterns, exploring the biological relevance of modules to the target traits, and identifying core genes in gene networks [53]. In this study, a weighted gene co-expression network was constructed via WGCNA based on 12 sets of transcriptome sequencing data, and co-expression modules that were highly significantly associated with oxalate were identified. The regulatory network of the blue module consisted of a set of genes controlling the carboxylic acid metabolism, including functions related to photosynthesis, Ca^2+^ binding, fructose 1,6-diphosphatase activity, and ATPase activity, suggesting the role of oxalate included regulating Ca homeostasis, photosynthesis, postharvest quality, and metal detoxification [28]. MD00G1109700, MD16G1191400, and MD01G1004300 genes in the blue module were related to photosystem I reaction center subunits, and MD09G1098400 acted as a cell cycle regulator. MD14G1070300 was shown to be involved in sucrose synthesis in the turquoise module. These identified genes may act as potential regulators of the CaOx biosynthesis pathway triggered by N. Similarly, studies have shown that Ca deficiency upregulated the expression of *WRKYs* in apple with bitter pit, and the overexpression of *MdWRKY75* significantly increased sucrose accumulation and the expression of *MdSWEET1* [1]. A significant proportion, 40.49%, of all DEGs included members of transcription factor families, including *Pkinase*, *WRKY*, *MYB*, and *AP2*. In the comparative analysis of sufficient Ca vs. low Ca and high N vs. low N, these transcription factors were shown to be predominantly upregulated. The regulatory mechanisms of upstream genes involved in oxalate synthesis have received limited attention, warranting follow-up study. The tomato NAC transcription factor *SlNAC63* negatively regulates the expression of *SlAAE3–1*, which is involved in oxalate degradation, to improve fruit quality [54]. In the future, it may be possible to use gene-editing technologies that reduce the oxalate content in edible plants and fruits to manipulate the abundance of anti-nutritive CaOx, and improve CaOx-based Ca bioavailability in crops, enhancing their nutritional quality.

## Conclusion

N has a drastic impact on Ca content, form, and distribution in apple plants. In this study, the balance model of the synergistic effect of N and Ca in apple leaves was first analysed. High N was shown to increase the CaOx content and proportion in the apple leaves. Electron microscopy and the analysis of Ca forms revealed that Ca was mostly deposited in the form of CaOx in phloem presenting a rhombic morphology. Then, FTIR confirmed that more carboxylates were produced in apple leaves under high N conditions. Specifically, high N greatly promoted oxalate synthesis by upregulating the **MdICL*, *MdOXAC**, and *MdMDH* genes, and inhibited oxalate degradation by downregulating the *AAE3* gene, thereby increasing oxalate accumulation in apple leaves. Additionally, transcriptome analysis confirmed that N and Ca affected genes involved in the regulation of photosynthesis, the TCA cycle, and the glycolytic flux, which are closely related to the oxalate biosynthesis. Following up on previous studies, our correlation analysis further clarified the mechanisms regulating CaOx formation under high N conditions via the photosynthesis/glycolysis – oxaloacetate –oxalate – CaOx pathway in apple leaves. Overall, these results provided transcriptome information with respect to the molecular mechanisms through which N regulates CaOx biosynthesis in apple leaves, as well as a theoretical foundation for an improved Ca-efficient apple cultivation management, and breeding of rootstocks with high Ca content.

## Methods

### Plant materials and growth conditions

The experiment was conducted at the Tai’an Experimental Station of Shandong Agricultural University (latitude 36°18′N, longitude 117°13′E) from May to July 2023. The apple plants used in this study, which belonged to the M9T337 apple dwarf rootstock, had approximately six leaves and a height of 10 cm and were planted on foam boards with seven holes (one hole was used for air stone ventilation). Each hole contained one plant, and each pot (sized at 28 × 21 × 9 cm) was supplied with 3.5 L of Hoagland nutrient solution [55]. Initially, the plants were cultivated with half-strength Hoagland nutrient solution for seven days to allow them to gradually adapt. Then, uniformly grown plants were selected for treatment. The apple rootstocks were subjected to a screening concentration experiment to determine the turning point of the synergistic interaction between N and Ca at concentrations of 10 mM and 5 mM, respectively [56]. The experiment was based on a double-factor random design, with two levels of Ca (0, 5 mM Ca^2+^) and two levels of N (0.05, 10 mM NO_3_^−^). The treatments were categorized as low N without Ca (L−: 0.5 mM NO_3_^−^ + 0 mM Ca), low N with Ca (L+: 0.5 mM NO_3_^−^ + 5 mM Ca), high N without Ca (H−: 10 mM NO_3_^−^ + 0 mM Ca), and high N with Ca (H+: 10 mM NO_3_^−^ + 5 mM Ca), and each was applied to 60 plants distributed across 10 pots. NaNO_3_ and CaCl_2_ served as the sources of NO_3_^−^ and Ca^2+^, respectively. Other specific nutrient concentrations included: 2 mM MgSO_4_, 1 mM KH_2_PO_4_, 0.1 mM Fe-EDTA, 37 μM H_3_BO_4_, 9 μM MnCl_2_·4H_2_O, 0.3 μM CuSO_4_·5H_2_O, 0.5 μM H_2_MoO_4_·H_2_O, 0.7 μM ZnSO_4_·7H_2_O, and 0.5 mM Ca(^15^NO_3_)_2_ (produced by Shanghai Institute of Chemistry, with an abundance of 10.14%, for ^15^N labeling), maintaining a pH of 6.0 ± 0.1 [55]. The nutrient solution was replaced every 3 days.

After 20 days of treatment, ^13^C pulse labeling was initiated using 0.2 g of Ba^13^CO_3_ (which had a ^13^C abundance of 98%). The labeling process involved placing the plants, labeling material, a fan, and reduced iron powder in a sealed labeling chamber made of clear film, and it was started at 9:30 a.m. by activating the fan. Simultaneously, plantlets located at a certain distance that were not contaminated by ^13^C were selected as blank controls to measure the natural abundance of this element. After 72 h of labeling, destructive sampling was conducted to measure ^13^C abundance [57].

### Analysis of photosynthetic parameters, leaf area, chlorophyll content, and rhizosphere pH

The photosynthetic parameters (i.e., *Pn*, *Gs*, and *Ci*) were measured between 9:00 a.m. and 11:00 a.m. using a CIRAS-3 portable photosynthesis meter (PP-Systems, Hertfordshire, UK). The fluorescence parameters were analysed using an FMS-2 portable pulse-modulated fluorometer. A Li-3100 leaf area meter (Li-COR, Inc., Lincoln, NE, USA) was used to measure the leaf area. Chlorophyll a and b were extracted from 1 to 3 new leaves (excluding the main vein) growing on the top and weighing 0.1 g using 95% ethanol. To determine the rhizosphere pH, apple roots that had been treated for 4 days were placed in a mixed culture liquid containing 0.01% bromocresol purple, 0.2 mM CaSO_4_, and 0.7% agar (pH = 6.5), and were kept in the dark for 45 min. Then, they were photographed.

### Nitro-blue tetrazolium (NBT) and 3, 3′-Diaminobenzidine (DAB) staining, and detection of antioxidant enzymes

NBT and DAB staining solutions were prepared at concentrations of 0.5 mg/mL 1.0 mg/mL, respectively. Then they were transferred into conical bottles and were added with pretreated plant tissue. They were maintained at a constant temperature of 28°C for 8 h in the dark. Once the staining was complete, the solutions were discarded, and the tissue was immersed in a fixative solution composed of ethanol, lactic acid, and glycerol at a 3:1:1 ratio. These solutions were heated for 5 min to remove all chlorophyll. Anhydrous ethanol was then added. Finally, the staining was evaluated.

For maleic dialdehyde (MDA) content analysis, 0.5–1 g of apple leaves was ground into a homogeneous paste using quartz sand and 2 ml of phosphate buffer. This mixture was then combined with 5 ml of a 0.5% thiobarbituric acid solution and heated up to the boiling point for 10 min. After cooling, the mixture was centrifuged at 12000 rpm for 15 min. Absorbance values were measured at wavelengths of 532 and 600 nm using an ultraviolet visible spectrophotometer (UV-2450). The MDA content was then calculated as follows: MDA content (μM/g FW) = 0.258 × (A532 − A600)/FW.

The H_2_O_2_ content, O_2_^−^ production rate, and catalase (CAT), peroxidase (POD), superoxide dismutase (SOD) activities were determined using the corresponding kits, which were purchased from Solarbio, and an ultraviolet–visible spectrophotometer.

### Measurement of N, Ca, and C contents

The plants were dried at 80°C until constant weight. Subsequently, they were homogenized using an electric grinder and sieved through a 0.25-mm sieve. A plant sample of 0.5 g was then weighed into a digestion tube, wetted with water, and treated with 5 mL of sulfuric acid overnight. The mixture was heated to 220°C for clarification and left boiling for 30 min to remove H2O2. The decocted sample was diluted to 50 mL in a volumetric flask, and its C and N contents were analysed using a Shimadzu TOC analyser. Total Ca content was measured using an atomic absorption spectrophotometer and calculated using the following equation: total Ca = C × V/M.

### Measurement of the net Ca^2+^ and NO_3_^−^ fluxes in mesophyll cells using NMT

An NMT Physiolyzer was used to measure the real-time rate of NO_3_^−^ and Ca^2+^ fluxes in and out of mesophyll cells. The leaf tissue was removed from the lower epidermis and cultured in a dish with test solution for 2–4 h, ensuring that the bottom surface was submerged. Subsequently, the test solution was discarded, the leaf tissue was carefully removed and fixed in a new dish, which was replenished with 5–10 mL of fresh test solution. The test solution contained 0.1 mM CaCl_2_, and 0.5 mM NO_3_^−^ for NO_3_^−^ flux (pH 6.0), and 0.1 mM CaCl_2_ for Ca^2+^ flux (pH 6.0). The target detection area was identified under a microscope, and the flux sensor was positioned at approximately 50 μm from the surface of the mesophyll tissue. Each sample was tested for 5–10 min, and each test was repeated six times to ensure reliable results. The flux data were obtained directly using the imFluxes V2.0 software (YoungerUSA LLC, Amherst, MA 01002, USA). A positive flux reading in mol cm^−2^ s^−1^ indicated efflux, while a negative value indicated influx.

### Analysis of ^15^N and ^13^C analysis

The seedlings labeled with ^15^N and ^13^C were deoxidized at 105°C for 30 min, then dried at 80°C to constant weight. The dry weight of each organ was measured, and the samples were then crushed by electric grinding, screened for 60 mesh, mixed, and bagged. The abundances of ^15^N and ^13^C were determined using a DELTAV Advantage isotope mass spectrometer (Thermo Fisher Scientific, Inc., USA) with three replicates.

### Measurement of Ca forms

The Ca forms were measured using the stepwise extraction method described in Song *et al.* [17]. In brief, a leaf sample of 0.5 g was separately extracted using distilled water, 1 mol/L sodium chloride, 2% acetic acid, and 5% hydrochloric acid prepared with distilled water. Each extract obtained from the above extractive liquids contained different Ca forms: water-soluble Ca (H_2_O-Ca), pectic Ca (NaCl-Ca), phosphate Ca (HAC-Ca), and CaOx (HCl-Ca), respectively. The Ca content in each extract was determined using an atomic absorption spectrophotometer.

### In situ distribution of N and Ca via scanning electron probe microscopy

Electron probe microanalysis was used to investigate the *in situ* distribution of N and Ca. The samples were collected from the fourth leaf down and the nodes attached to the stem (including the stem and petiole with the leaf) of plants subjected to different treatments. These samples were promptly prepared, made conductive, and observed using a JXA-8100 scanning electron microscope equipped with an X-ray spectroscope. The electron probe had an acceleration voltage of 20 KV and an emission current of 2 × 10^−8^ A. Additionally, a secondary electron detector was employed for sample detection. The distribution of N and Ca in the tissues was visualized as small white spots on a black background, with their density and brightness indicating the nutrient contents. The two maps obtained were merged using Photoshop 2020 to pseudo-color the secondary electron images and the X-ray Ca signal localization map, enhancing the visualization of the *in situ* Ca distribution.

### Histological observation of CaOx crystals

Fresh leaves were cut into 5 mm sections and fixed with a FAA fixative for 24 h. Subsequently, they were washed several times with 50% alcohol. The histological samples were pretreated following the protocol described in Huang *et al.* [36]. In brief, the samples were cut into slices with a thickness of 6–8 μm using a Leica RM 2235 rotary microtome. These slices were then attached to microscope slides, stained with safranin, and observed and photographed using a Leica DMLB microscope.

### FTIR analysis

Leaf samples obtained from different treatments were collected and dried, then they were crushed using a high-speed grinder and then transferred to an agate mortar for further grinding. Once uniformly ground, each sample was pressed into a tablet and subjected to testing using a Fourier infrared spectrometer (Germany BRUKER). The testing was conducted in triplicate to ensure accuracy. The collected infrared spectroscopic information was processed in Omnic9.0 to determine absorption levels, conduct automatic baseline calibration and smoothing, and quantify the second-order derivatives.

### Determination of the intermediates in glycolysis and the TCA cycle

Sucrose and fructose contents were determined using a sucrose content detection kit for plant tissues (Solarbio, BC2465). The contents of malate, oxaloacetate, ascorbic acid, and citrate content were determined via HPLC using a Carbopac PA-1 column (Dionex Corp., Sunnyvale, CA, USA). Glyoxylate reacted with phenyl hydrazine to form phenylhydrazone, and then the derivative was quantified via HPLC. A mobile phase solution containing 0.5% KH_2_PO_4_ and 0.5 mM tetrabutylammonium hydrogen sulfate buffered at pH 2.0 with orthophosphoric acid was used to determine the oxalate content. The flow rate was 1 mL min^−1^, and the compound was detected at 220 nm.

### Transcriptome analysis and qRT-PCR assays

Apple plants were treated with different N and Ca concentrations for 4 days. The leaves were removed from liquid N storage and then ground into a powder. Then, three samples per treatment (containing six plants each) were sent to Novogene Bioinformatics Technology Co., Ltd (Beijing, China) for RNA extraction and transcriptome sequencing and analysis. RNA was extracted from apple leaves for sequencing, and after screening the raw data, the sequencing error rate and GC content distribution were examined to obtain clean reads for subsequent analyses. The FPKM values were used to indicate transcript or gene expression levels (Fig. S1, see online supplementary material).

A 20-μL reaction system was used for qRT-PCR analysis. The total reaction volume included 10 μL of SYBR Green Supermix (TaKaRa), 7 μL of double distilled water, 1 μL of cDNA template, and 1 μL of each forward and reverse primer. Relative expression was quantified using the 2^-∆∆Ct^ method, and *MdActin* was selected as the internal reference gene. All primers are listed in Table S1 (see online supplementary material).

### Statistical analysis

The obtained data were analysed using GraphPad Prism 9 and Microsoft Excel 2016 and were expressed as mean ± standard deviation. Bioinformatics analysis was performed in *R* (*R* v.4.1.1). The network correlation diagram was visualized using Gephi (v.0.9.2).

## Supplementary Material

Web_Material_uhae208

## Data Availability

The authors confirm that all experimental data are available and accessible via the main text and/or the supplemental data.
