# Supplementary material for: Nitrogen reduces calcium availability by promoting oxalate biosynthesis in apple leaves
Source: Hortic Res. 2024 Jul 30;11(10):uhae208. doi: 10.1093/hr/uhae208 (PMC11450213; doi:10.1093/hr/uhae208)
Supplement: Web_Material_uhae208 [file web_material_uhae208.zip › Supplementary material.docx]

**Supplementary materials**

Table S1 Primers used for qRT-PCR

| Gene ID | Left Primer | Right Primer |
| --- | --- | --- |
| MD14G1102200 | TCGACGTTTTGGTCCGACAT | CCCTCCGCGATTAATCAGCT |
| MD06G1079100 | GACATGGTCGCATACAACGC | GGCAACCTCTGGATGTGACA |
| MD05G1126300 | TTCTCAAGGAGAGCTTGCCG | GCATCCTTGAGCCTTTCCCT |
| MD04G1191900 | AGAGAACGTTGCGAGCATGA | CAGAAGTATCGAGGGTCGGC |
| MD14G1092700 | AGTTCAAGGAGGCCTTCAGC | TGGCCATCATCACCTTGACA |
| MD11G1183900 | CGAGTTCAAGGAGGCGTTCA | TGGCCATCATGACTTTGACGA |
| MD08G1206900 | CGCCAAGGTGAAGTTTGCTC | CACGGCACCATCATAACCCT |
| MD15G1153500 | CGAGCTCTTCGCCAAAATCG | GACTGGATCTTCTCGCTCGG |
| MD10G1258700 | ATTACGTTGCCCCTGAGGTG | ATGATCTCATTAGCCGGGCG |
| MD05G1140300 | GGCCTTCAGACCGCTTAACT | TTGCCCTTTTGCATCATGGC |
| MDActin | TGACCGAATGAGCAAGGAAATTACT | TACTCAGCTTTGGCAATCCACATC |

Table S2 Difference and enrichment analyses statistics

| Groups | All | Up | Down | GO | KEGG |
| --- | --- | --- | --- | --- | --- |
| H+ vs L+ | 441 | 276 | 165 | 371 | 61 |
| H- vs L- | 7164 | 3065 | 4099 | 946 | 115 |
| L+ vs L- | 1028 | 619 | 409 | 405 | 72 |
| H+ vs H- | 3352 | 2222 | 1130 | 828 | 107 |

“All” means the number of all DEGs; “Up” means the number of up-regulated DEGs; “Down” means the number of down-regulated DEGs; “GO” means the number of GO terms; “KEGG” means the number of pathways.


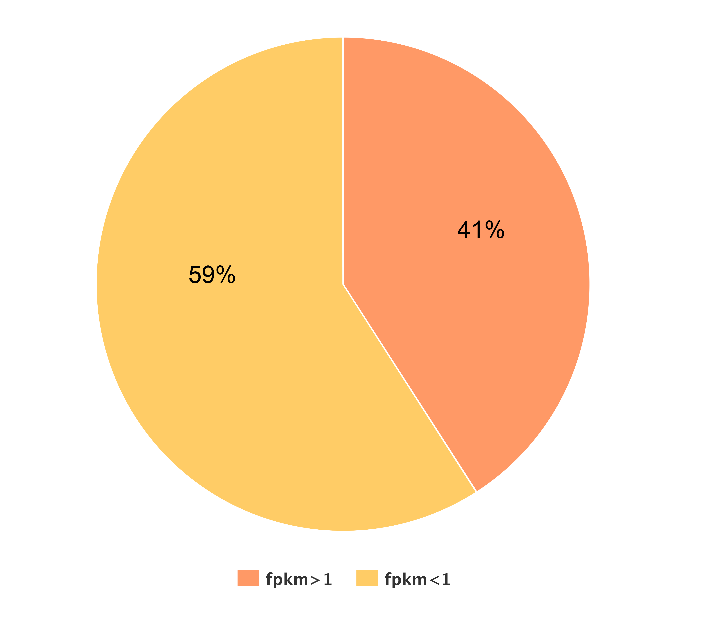


Fig. S1. Gene expression levels in apple leaves under nitrate and calcium conditions.


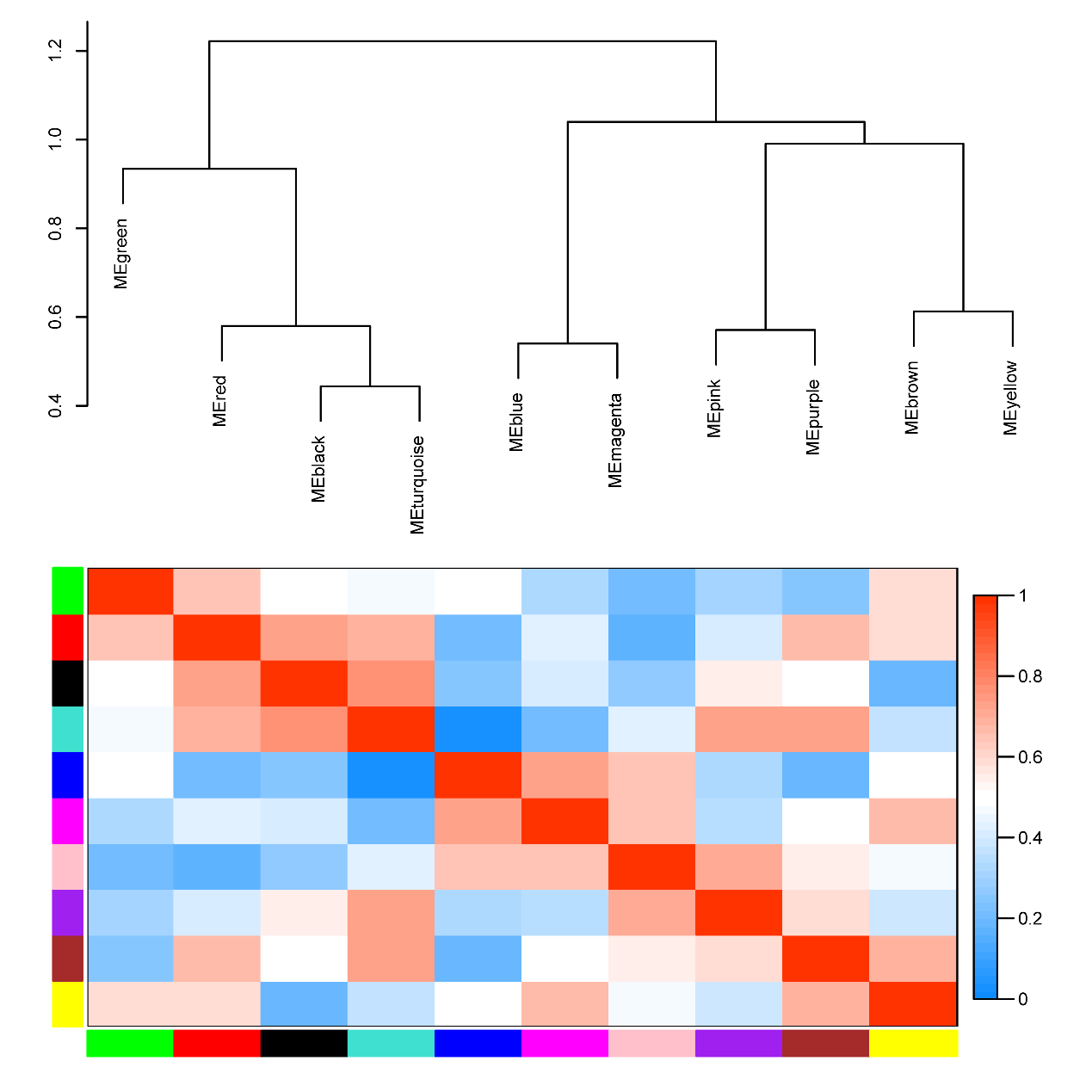


Fig. S2. Correlation analysis of co-expression modules.

Red means high correlation, while blue means low correlation.


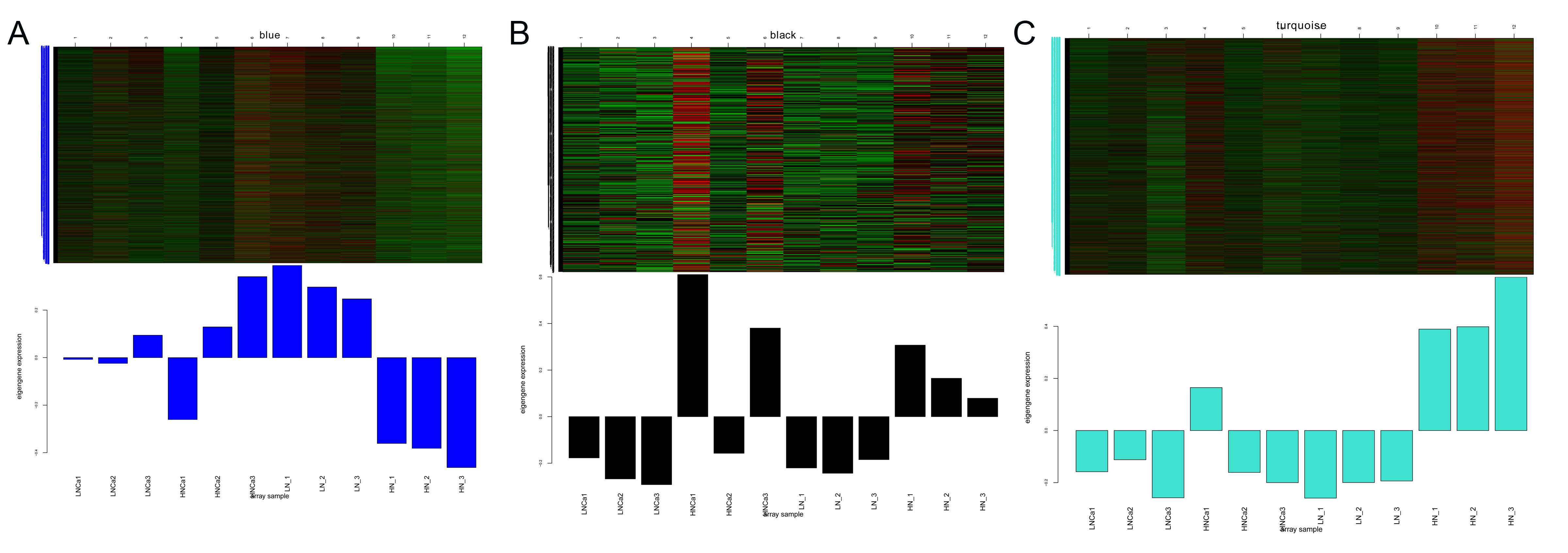


Fig. S3. Gene expression profiles of the key co-expression modules.


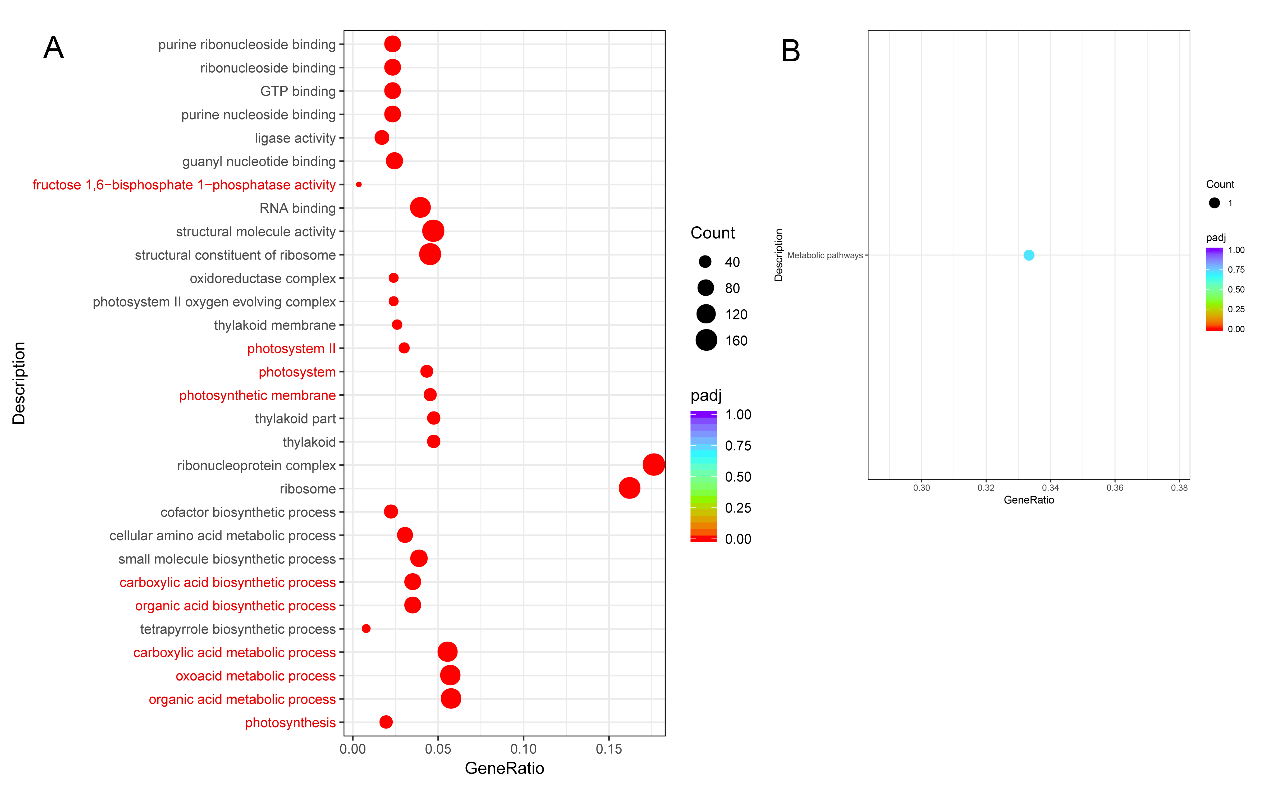


Fig. S4. GO and KEGG enrichment analyses of genes of blue module.

1. The top 30 most enriched GO terms. (B) Enriched KEGG pathways.


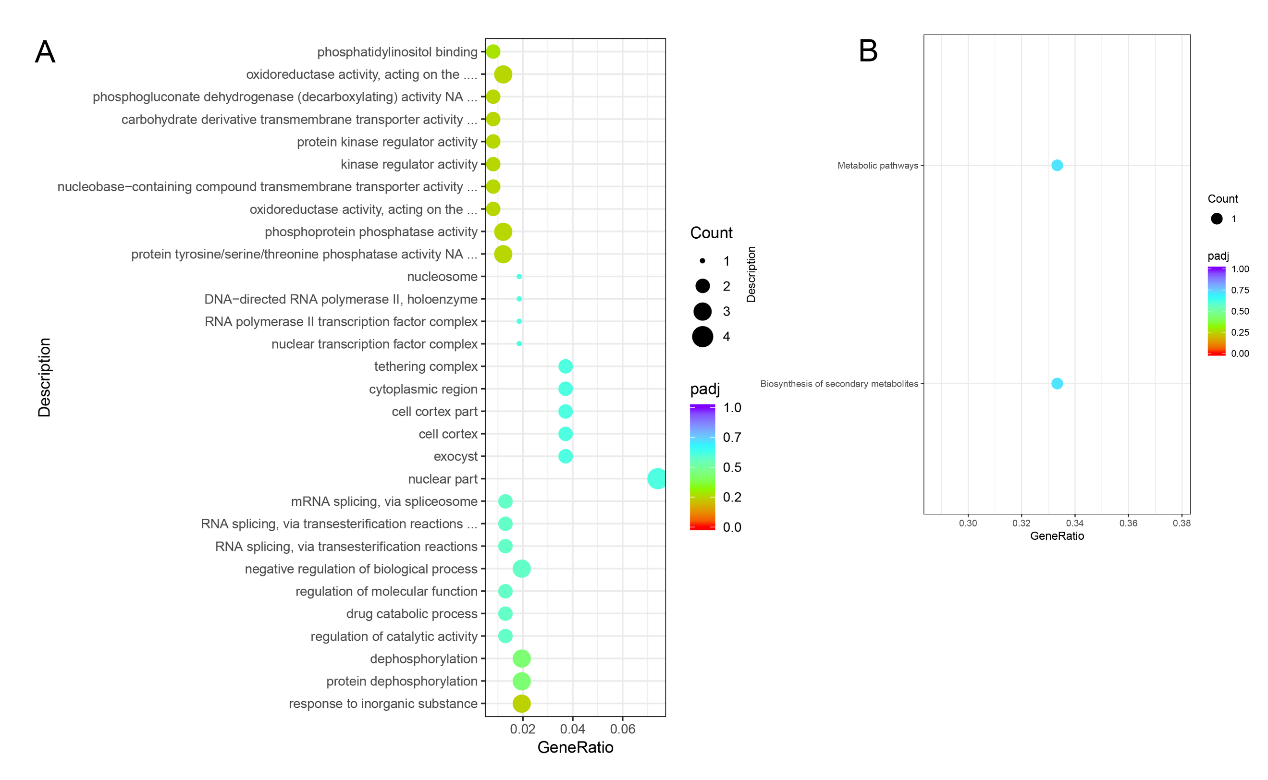


Fig. S5. GO and KEGG enrichment analyses of genes of black module.

(A) The top 30 most enriched GO terms. (B) Enriched KEGG pathways.


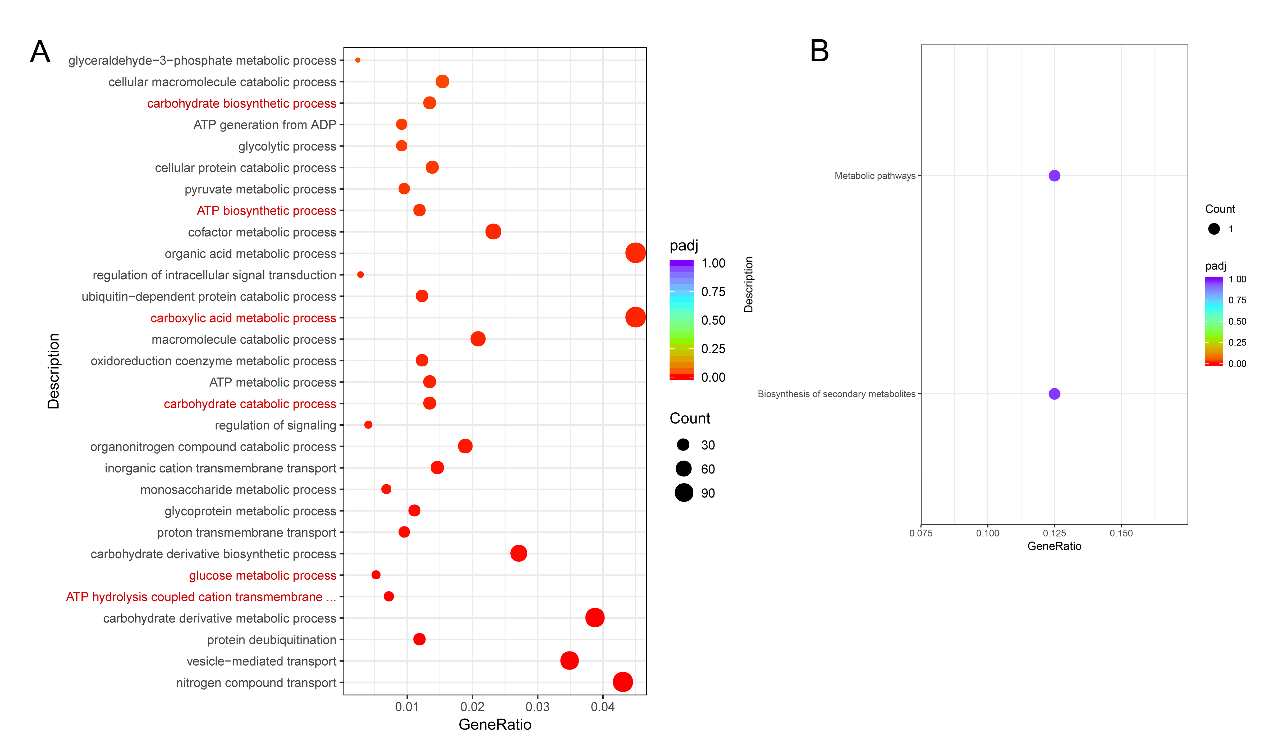


Fig. S6. GO and KEGG enrichment analyses of genes of turquoise module.

(A) The top 30 most enriched GO terms. (B) Enriched KEGG pathways.


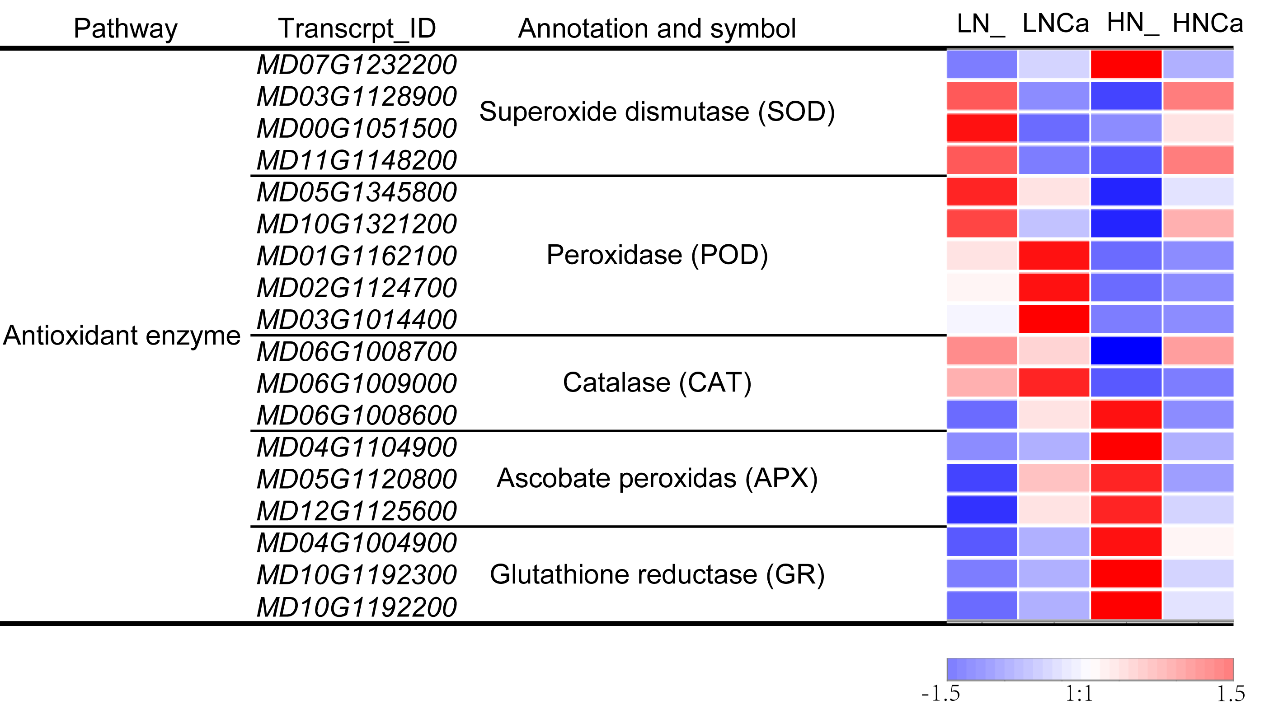


Fig. S7. Heatmap of antioxidant enzyme-related gene expression


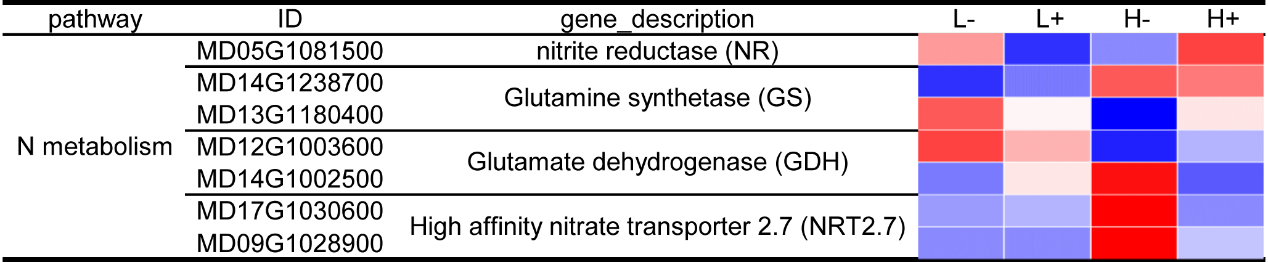


Fig. S8. Heatmap of nitrogen-related gene expression


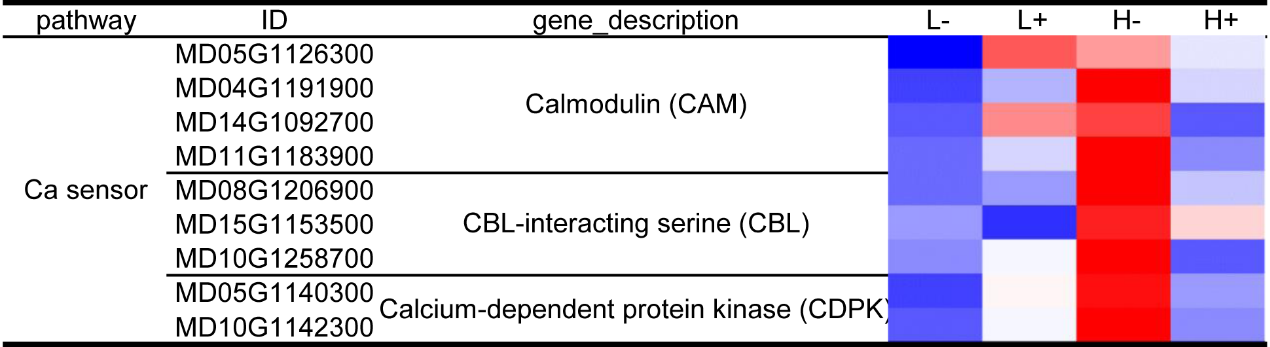


Fig. S9. Heatmap of calcium-related gene expression


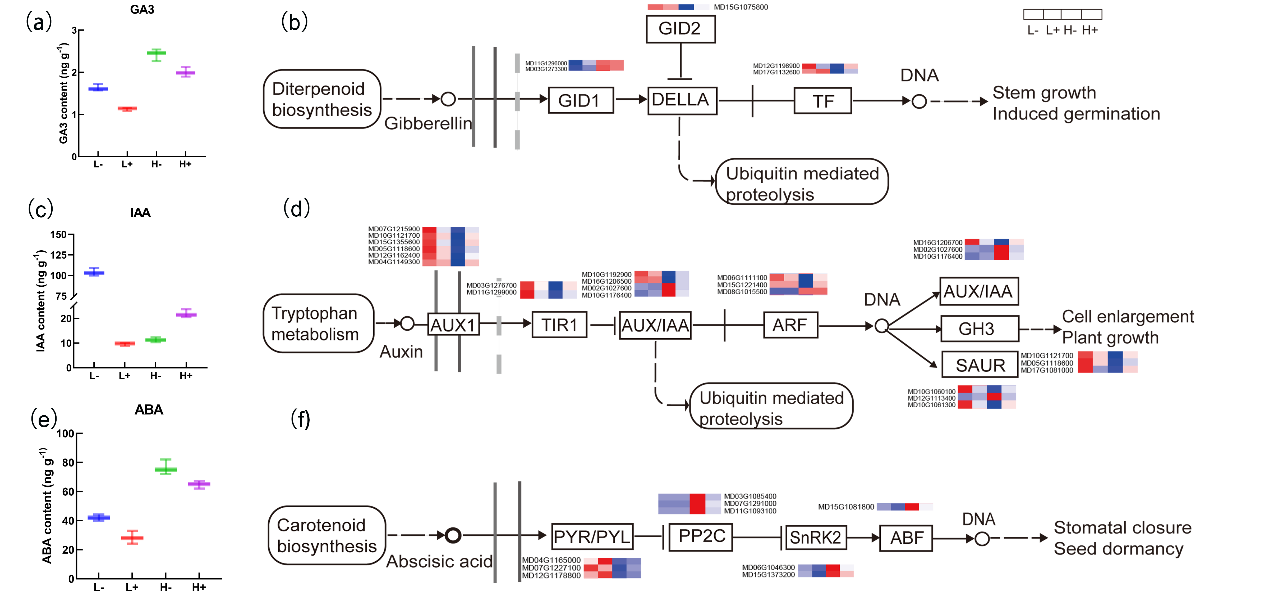


Fig. S10. Plant hormones involved in NO_3_^-^ and Ca^2+^ response. (a) Changes in GA3 content. (b) GA3 signal transduction pathways and gene expression patterns. (c) Changes in IAA levels. (d) IAA signal transduction pathways and gene expression patterns. (e) Changes in ABA content. (f) ABA signal transduction pathways and gene expression patterns. The bars represent the mean ± standard error (n = 3).
